# Intracellular Vesicles as Reproduction Elements in Cell Wall-Deficient L-Form Bacteria

**DOI:** 10.1371/journal.pone.0038514

**Published:** 2012-06-06

**Authors:** Yves Briers, Titu Staubli, Markus C. Schmid, Michael Wagner, Markus Schuppler, Martin J. Loessner

**Affiliations:** 1 Institute of Food, Nutrition and Health, ETH Zurich, Zurich, Switzerland; 2 Department of Microbial Ecology, University of Vienna, Vienna, Austria; University of Illinois at Chicago College of Medicine, United States of America

## Abstract

Cell wall-deficient bacteria, or L-forms, represent an extreme example of bacterial plasticity. Stable L-forms can multiply and propagate indefinitely in the absence of a cell wall. Data presented here are consistent with the model that intracellular vesicles in *Listeria monocytogenes* L-form cells represent the actual viable reproductive elements. First, small intracellular vesicles are formed along the mother cell cytoplasmic membrane, originating from local phospholipid accumulation. During growth, daughter vesicles incorporate a small volume of the cellular cytoplasm, and accumulate within volume-expanding mother cells. Confocal Raman microspectroscopy demonstrated the presence of nucleic acids and proteins in all intracellular vesicles, but only a fraction of which reveals metabolic activity. Following collapse of the mother cell and release of the daughter vesicles, they can establish their own membrane potential required for respiratory and metabolic processes. Premature depolarization of the surrounding membrane promotes activation of daughter cell metabolism prior to release. Based on genome resequencing of L-forms and comparison to the parental strain, we found no evidence for predisposing mutations that might be required for L-form transition. Further investigations revealed that propagation by intracellular budding not only occurs in *Listeria* species, but also in L-form cells generated from different *Enterococcus* species. From a more general viewpoint, this type of multiplication mechanism seems reminiscent of the physicochemical self-reproducing properties of abiotic lipid vesicles used to study the primordial reproduction pathways of putative prokaryotic precursor cells.

## Introduction

Bacteria display great adaptability in response to a changing environment, and can undergo dramatic phenotypic shifts to assure their survival under a variety of unfavorable conditions. A quite drastic response is the (partial) loss of the cell wall, which occurs when a bacterium is exposed to compounds interfering with cell wall integrity or synthesis (e.g., antibiotics, lytic enzymes, amino acids), or as a strategy to escape phage predation or killing by the immune system [1]. Interestingly, bacteria are able to survive despite loss of the cell wall, and even continue to propagate provided that osmotic protection is present [2]. These viable and actively reproducing cell wall-deficient bacterial derivatives have commonly been referred to as L-forms. Their emergence and occurrence has been reported for several Gram-positive and Gram-negative bacterial species. Although conversion to the L-form state may be considered a universal and widespread property of bacteria, it is only poorly understood [1]–[3]. Cell wall-deficiency may be induced *in vitro,* by exposure to sublethal doses of cell wall-active antibiotics such as β-lactams, but may also occur *in vivo* [4]. In order to perform cell division, L-forms must be able to compensate for the lack of an organized cell wall structure, and for the consequent inability to undergo a typical binary fission. Several alternative reproduction strategies have been described in the older literature, such as extracellular budding, binary fission-like processes, internal fragmentation, or the hypothetical formation of small elementary bodies [5], [6]. More recently, an FtsZ independent extrusion-resolution based division mechanism was reported for *Bacillus subtilis* L-forms. Protrusions elongate from their cell membrane and resolve into pleomorphic bodies assumed to represent the progeny [7]. This diversity in alternative reproduction mechanisms and pathways illustrates the surprisingly large plasticity of the bacterial cell in absence of a cell wall.

We have previously described an L-form model system for the Gram-positive pathogen *Listeria monocytogenes* [8]. Transcriptome analysis indicated induction of stress-associated genes as the cells need to adapt to the unusual cell wall-deficient life style, while transport and metabolism functions were attenuated. Larger L-form cells feature intracellular vesicles and led to the intriguing hypothesis that these might represent the reproduction units. Our current work demonstrates that *L. monocytogenes* L-forms contain multiple nucleoids per cell and retain the full genetic potential for peptidoglycan synthesis. Employing specific dyes, Raman microspectroscopy, and confocal time-lapse microscopy, we provide additional support for the model that L-form cells are able to propagate and release viable daughter vesicles, and show that this type of intracellular budding mechanism appears to be also used by other members of the Firmicutes.

## Results

### *Listeria Monocytogenes* L-forms are Multinucleated Cells

The relatively strong fluorescence of *L. monocytogenes* L-forms stained with the DNA dye DAPI [8] suggested the presence of more than one bacterial chromosome inside a single L-form cell. We determined the average chromosome number per cell by quantification of a single-copy gene, followed by averaging over a population of L-form cells. Since determination of L-form colony forming units was not possible (no growth on agar plates), cell counts were based on using an optical Helber microscope slide counting chamber. To determine the number of chromosomal copies per sample, a 121 bp fragment of the single *actA* gene was amplified using quantitative real-time qPCR. For 3 independent samples, we measured an average of 18.0±3.6 genome copies per cell. In parental (walled) cells, an average of 1.9±0.5 bacterial chromosomes per cell was found. These data demonstrate that *L. monocytogenes* L-forms contain multiple nucleoids with about a 10-fold increase of chromosome copies in comparison to the normal cell-wall proficient bacteria.

### *Listeria* L-form Transition does not Require Predisposing Mutations

The L-form strain used in this study has been cultured and sequentially propagated for more than 5 years. It is stable and autonomous, i.e., able to divide and multiply indefinitely, and does not revert to the walled state [8]. Previously, stable L-forms were thought to result from genetic changes or mutation, leading to invariable defects in the peptidoglycan synthesis machinery [2], [9]–[10]. To investigate the situation in *Listeria monocytogenes*, the complete genome sequence of the L-form strain used here has been determined by Illumina-based resequencing, yielding a 57-fold coverage of the reference genome [11]. Assembly and comparison with the genome of bacterial parent indicated that all genes involved in peptidoglycan precursor synthesis, formation and recycling of the peptidoglycan network, and cell division were unchanged in the L-form genome. Only two single-nucleotide polymorphisms were detected, 51 nt upstream of a putative IIAB component of a putative mannose-specific phosphotransferase system (PTS), and within *lmo0588*, a putative deoxyribopyrimidine photolyase. We also found a 34 nucleotide deletion in a putative HMG-CoA synthase gene (see Text S1). These changes were confirmed by independent Sanger sequence analysis. However, screening of these loci in several other stable L-form lines derived from *L. monocytogenes* Scott A generated throughout this work did not confirm these alterations, i.e., did not yield any evidence that these changes represent predisposing mutations. We conclude that although different minor genetic alterations might occur, they are irrelevant for L-form transition and stability.

### L-form Cells Accumulate Intracellular Vesicles

**Figure 1:**
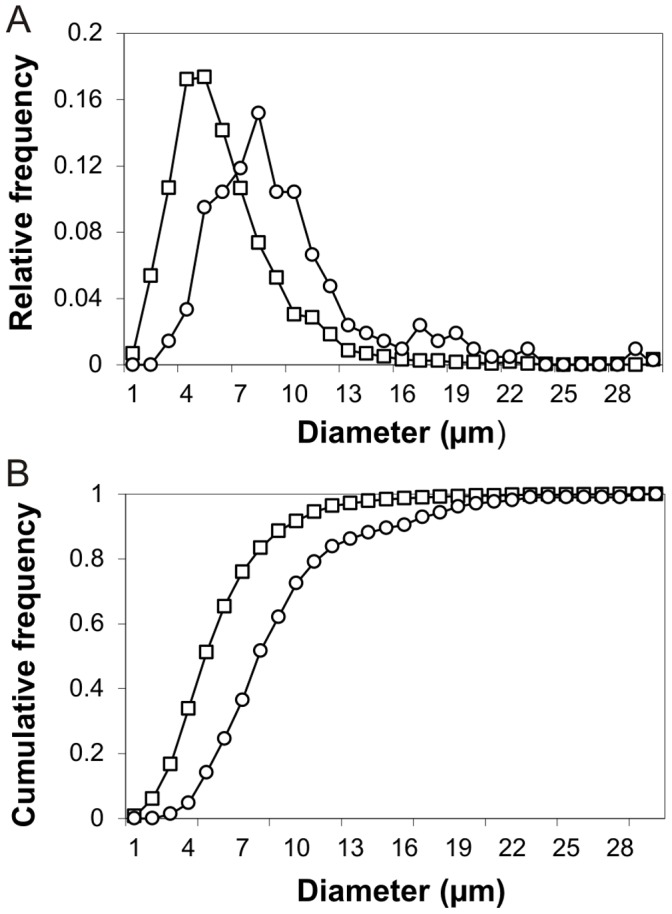
Morphological features of *L. monocytogenes* L-forms. (A) The size distribution of L-forms (n = 2801) (squares) and its subpopulation containing intracellular vesicles (n = 211) (circles) is represented as the relative frequency per 1 µm size classes. Cells from the whole population have an average diameter of 6.1±3.1 µm. L-forms containing intracellular vesicles have an average diameter of 9.4±4.4 μm. (B) The subpopulation with intracellular vesicles was divided in a group with 1, 2 or 3 intracellular vesicles (squares) and a group with 4 or more intracellular vesicles (circles). Cumulative frequency curves are shown for both groups in incremental steps of 1 µm. Four or more intracellular vesicles have been mainly accumulated by the larger L-form cells.

In soft agar media, L-form cells grow to distinct colonies, which consist of a core of mostly cell debris, surrounded by multiplying L-form cells in the peripheral zones (Figure S1). In contrast to the uniformly rod-shaped parental cells, L-forms are spherical and feature a highly variable size. Cells diameter shows an asymmetrical right-tailed distribution, ranging from 0.5 to 30 µm, with an average of 6.1±3.1 μm (n = 2801) (Figure 1A). To compare the volume of L-form cells with parental bacteria, dimensions of *L. monocytogenes* Scott A grown for 18 h (BHI, 30°C) were measured (n = 20). The rods of average length 2.3±0.4 µm and width 0.7±0.1 µm represent cylinders, with an average volume of 0.89 µm^3^. L-forms are largely spherical bodies, and the volume of an average vs. the largest L-form cells observed is 119 vs. 14,137 µm^3^, which corresponds to a volume increase of approximately 134 and 16,000 compared to the parental cells. The L-form subpopulation with intracellular daughter vesicles (n = 211, corresponding to 7.5% of the total) feature larger cells, with an average diameter of 9.4±4.4 μm. A distribution plot shows that intracellular vesicles accumulate mostly, but not exclusively, in the larger L-forms (Figure 1A). The subpopulation of L-forms containing intracellular vesicles was further subdivided in groups with up to 3, or 4 and more intracellular vesicles. Comparison of the cumulative frequency curves of both groups indicate that the number of intracellular vesicles further increases when mother cells grow larger (Figure 1B). Altogether, growing L-form cells continue to increase in size, and the larger cells may accumulate up to several tens of intracellular vesicles, which then assume most of the cytosolic volume of the mother cell. These vesicle-rich cells are most frequently observed among the distal, outermost regions of a soft-agar grown colony (Figure S1), which is consistent with the idea that L-form multiplication and colony expansion takes place at the periphery.

### Genesis of Daughter Vesicles is Accompanied by Encapsulation of Cytoplasmic Material

**Figure 2:**
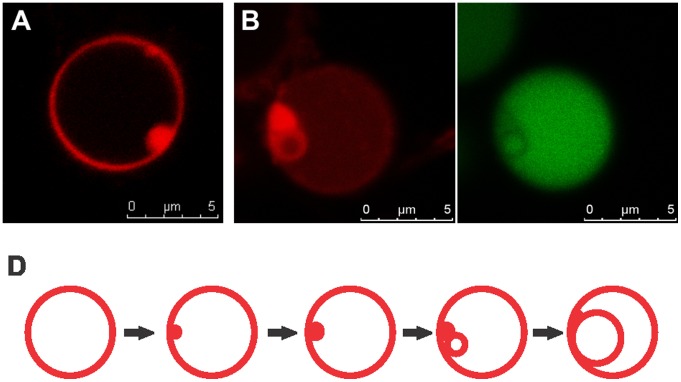
Origin of intracellular vesicles. (A) First, uniformity of the unilamellar membrane structure is disturbed and phospholipids accumulate along the membrane. (B) Small intracellular vesicles are always generated in direct proximity to these lipid domains (left), and cytoplasmic material of the maternal cell is encapsulated into the vesicles as visualized by the compartmentalization of GFP (right). Membranes stained with CellTrace BODIPY TR methyl ester and cytoplasmic GFP fluorescence are shown in red and green, respectively. All images are confocal. (D) Current model for vesicle genesis. Lipid accumulations act as a pool for incorporation of phospholipids into a membrane structure, resulting in enlargement of intracellular progeny vesicles by a self-organizing, spontaneous process typical for bipolar phospholipids.

To provide more insight into the possible origin and development of intracellular vesicles, we focused on the smallest size vesicles. With the aid of a membrane stain, many sites of disrupted uni-lamellarity and phospholipids accumulation could be observed along the mother cell membranes (Figure 2A). Small intracellular vesicles were always located in close proximity to these lipid accumulations (Figure 2B). Their inside consisted of an apparently normal mother cell cytoplasm, as visualized by the compartmentalization of cytosolic GFP (Figure 2C) and other proteins and nucleic acids (see below). We propose that these lipid accumulations represent the phospholipid pool and nucleation site for the generation of new vesicles. This process appears to be largely self-organizing and spontaneous, similar to the formation of liposomes. Subsequent enlargement of the vesicles may be supported by the existing lipids from the focal accumulations, or newly synthesized phospholipids from the mother cell (Figure 2D). Based on our observations, we also suggest that the development of new, small intracellular vesicles occurs in a random fashion, as the biogenesis of different intracellular vesicles often takes place simultaneously at apparently random sites along the mother cell membrane.

### Intracellular Vesicles as Viable Reproduction Elements

**Figure 3:**
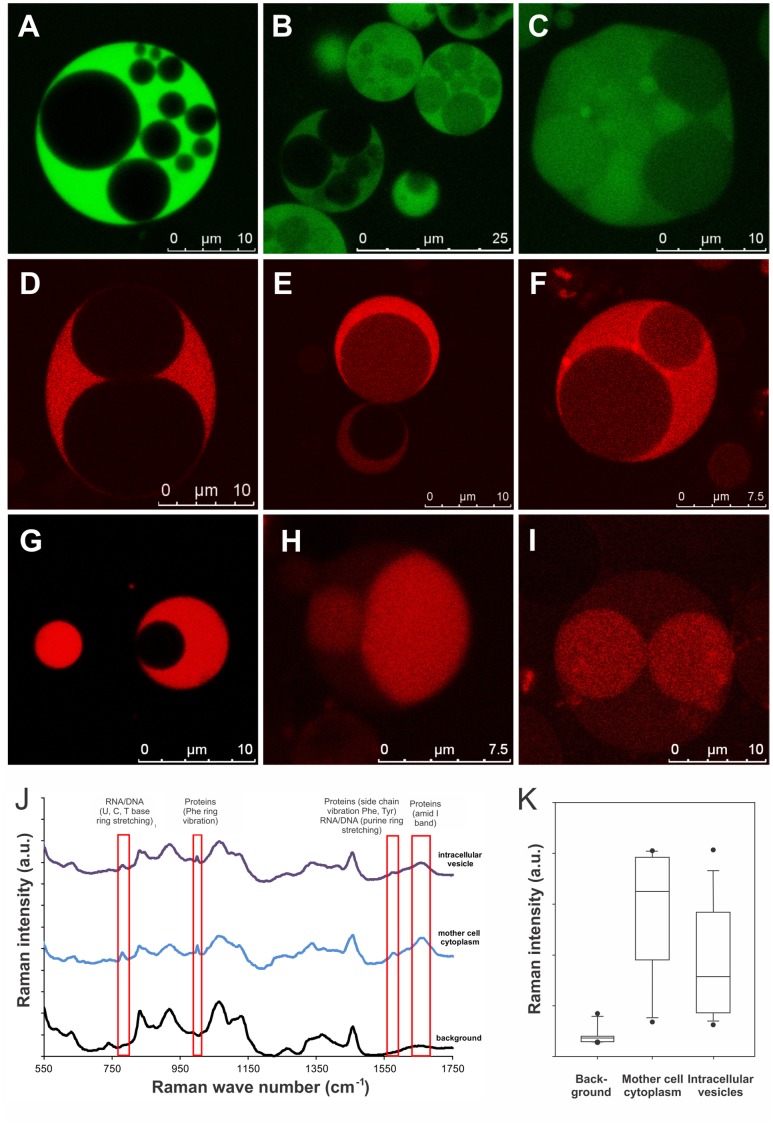
L-form cells feature intracellular viable progeny vesicles. The viability of progeny vesicles was assessed by GFP synthesis and fluorescence (A–C), spatial distribution of Rho123 (D–F), and reductive metabolism of a tetrazolium dye (G–I). Intracellular vesicles either showed no signals, faint signals, or strong signals, in some cases stronger than the mother cell. Panel J shows baseline corrected and normalized Raman spectra of the average background control (lower curve, n = 10), mother cell cytoplasm with a strong GFP signal (middle curve, n = 13) and non-fluorescent large intracellular vesicles (upper curve, n = 18). The different spectral curves have been offset but the scale was retained. Relevant peaks are indicated by red boxes, and correspond to vibration signals from RNA/DNA (785 cm^−1^, 1583 cm^−1^), and proteins (1003 cm^−1^, 1583 cm^−1^ and 1666 cm^−1^). These molecules are present both in the mother cell cytoplasm and, albeit less pronounced, inside the intracellular progeny vesicles. Panel K shows a box plot of Raman intensities measured for the background (buffer), mother cell cytoplasm, and intracellular vesicles at 1666 cm^−1^, which reflects protein content. The median intensity is lower in intracellular vesicles compared to the mother cell cytoplasm, indicating a slightly lower protein content in the progeny vesicles.

We monitored viability and metabolic processes of the intracellular vesicles using several reporters. Synthesis and maturation/oxidation of GFP was used as an indicator for active transcription and translation processes (Figure 3A-C). Spatial distribution of the cationic membrane-permeable dye Rhodamine123 (Rho123) served as indicator for an existing membrane potential, as well as charge and polarity of intracellular vesicle membranes [12]. Rho123 is only retained in compartments with a “negative inside” membrane potential, and distributes across lipid compartments according to the highest polarity (Figure 3D-F). In addition, metabolic reduction of a colorless tetrazolium salt into a red formazan dye was used as indicator for respiratory activity and oxidative metabolism (Figure 3G-I) [12]. While a majority of the intracellular vesicles did not appear to be positive for these indicators, others showed clear signs of respiratory and metabolic activities, and presence of a membrane potential. This suggests that only a fraction of the intracellular vesicles inside the vesiculated L-forms may be viable and active, while most of them seem to be inactive and show no attributes of life.

We employed confocal Raman microspectroscopy to fingerprint the molecular composition of intracellular vesicles. The confocal configuration of the spectroscope and the selection of intracellular vesicles to feature a diameter of at least twice the thickness of an optical slice assured that only an internal volume within the intracellular vesicle would be analyzed, excluding interference from the surrounding maternal cell cytoplasm. Spectra were obtained from maternal cell cytoplasm (n = 13), and non-fluorescent intracellular daughter vesicles (n = 18). As a control and reference, background spectra were used (n = 10). Comparison of the spectral data of the mother cell cytoplasm with the unspecific background enabled identification of unique spectral regions, representing nucleic acids (785 cm^−1^ and 1583 cm^−1^) and proteins (1004 cm^−1^, 1583 cm^−1^ and 1666 cm^−1^) [13]. Similar peaks were recorded from the intracellular vesicles (Figure 3J). A box plot comparison of Raman intensities of the most prominent peak at 1666 cm^−1^ (indicative for protein amide bonds) of the background, mother cell cytoplasm, and large intracellular vesicles indicated protein content in the daughter vesicles was lower than in the mother cell cytoplasm, but significantly higher than the background (Figure 3K). The large variation in intensities measured for mother cell cytoplasm and intracellular vesicles reflects a high heterogeneity.

Altogether, the above findings indicate that intracellular vesicles contain all essential biomolecules such as proteins and nucleic acids, but only a fraction of them features metabolic activity and respiration when still contained within the mother cell.

### Activation of Progeny Occurs upon Release from the Mother Cell

**Figure 4:**
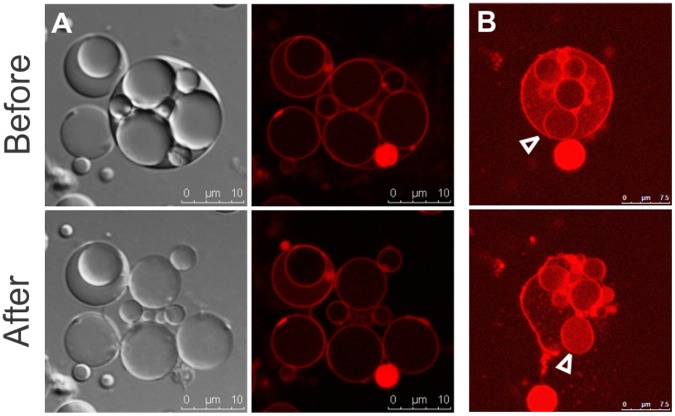
Release of internal vesicles. (A) Following collapse of an L-form mother cell, intact progeny cells are released. Membranes are visible by using DIC (Differential Interference Contrast) microscopy (left); dye-labeled membranes (right) are shown immediately before (upper) and after the collapse (lower). (B) A vesiculated cell labeled with Rho123 (upper) disintegrates and releases a vesicle (lower image, arrow head). The increase in the Rho123 signal on the surface of the released vesicle suggests an increase in membrane charge.

**Figure 5:**
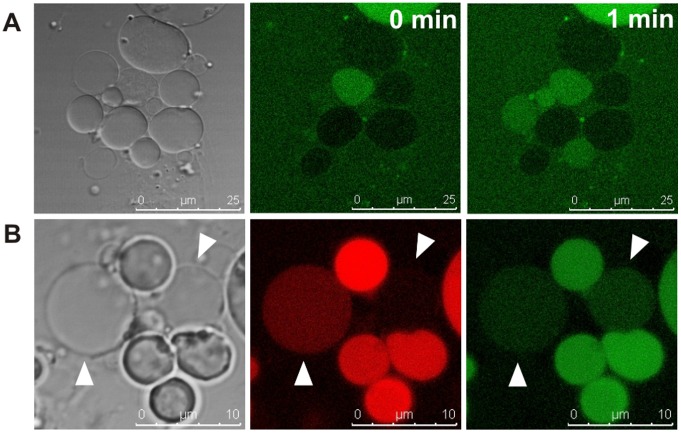
Activation of progeny cells upon release. Panel A: A large mother cell, which collapsed immediately before the first image was recorded, is shown. At this time point (0 min), only one of the released vesicles displays faint GFP fluorescence, whereas after 1 minute three more vesicles display a clear GFP signal. Panel B: Freshly released daughter vesicles (arrow heads) typically show low phase contrast (left), and weak CTC metabolism (middle) and GFP fluorescence (right), which increases shortly thereafter.

Unexpectedly, most of the intracellular vesicles released into the medium by collapse of the mother cell remain intact (Figure 4A). This process is often accompanied by an increase in Rho123 fluorescence, indicating an upshift in membrane potential upon release (Figure 4B). This suggested that intracellular vesicles are “activated” upon release from the mother cell, as shown in Figure 5A. Initially, only one of the daughter vesicles released from of a collapsed mother cell shows a green fluorescent GFP signal, while three others appear shortly thereafter, within approximately 1 minute. The delay might be due to the requirement for oxidation of the immature fluorophores of pre-synthesized, non-fluorescent GFP molecules, rather than *de novo gfp* transcription and translation, and may also be influenced by a pH shift within the vesicles after release from the cytoplasmic compartment. Freshly released vesicles frequently feature (initially weak) signals from both CTC reduction and GFP fluorescence (Figure 5B).

In conclusion, intracellular lipid vesicles in cell wall-deficient *Listeria* L-forms appear to represent viable reproductive elements, and most of them show signals of cellular viability only after release from the mother cell.

### Activation of Daughter Cells following Depletion of Mother Cell Membrane Charge

**Figure 6:**
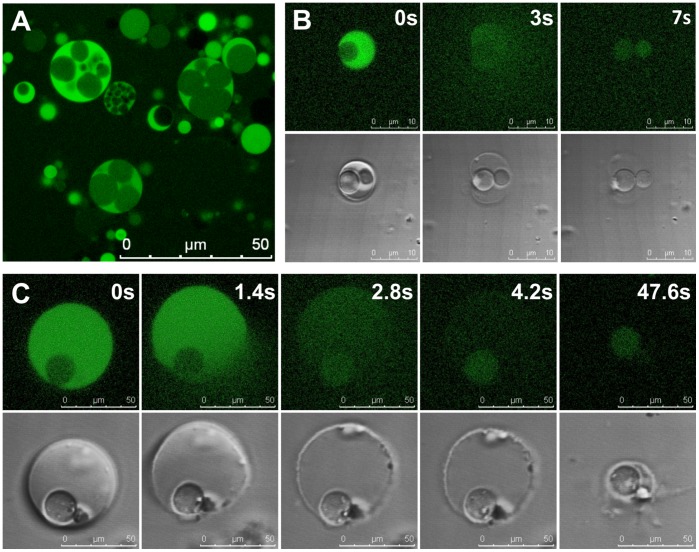
Membrane disruption triggers release and activation of daughter cells. Panel A: Brief exposure of large, vesiculated L-form cells to nisin induces membrane permeability. The subsequent occurrence of green fluorescent signals from intracellular vesicles indicates preliminary activation of daughter cells. Panels B, C: Nisin also promotes disintegration of mother cell membranes, followed by release of the vesicles (compare Movie S1).

An explanation for the fact that most of the daughter cells remain silent until their release is that creation of charge and polarity across the daughter cell membrane is counteracted as long as the surrounding mother cell maintains its own respiratory activity and membrane potential. However, a fraction of the internal vesicles can overcome this barrier and show activity while still being enclosed within the maternal cytoplasm and charged membrane. We tested this hypothesis using the pore-forming lantibiotic nisin to trigger depolarization and subsequent destruction of the maternal cytoplasmic membrane. Nisin is a small (34 aa) peptide which binds to lipid II molecules [14], abundant in L-form membranes [8]. It inserts into the membranes, oligomerizes, and forms pores, resulting in an unspecific efflux of ions and small molecules including ATP [15]. Pulse-challenge of L-form mother cells using low nisin concentrations (185 nM) strongly increased the frequency of green fluorescent intracellular vesicles, indicating a role of membrane charge for viability of daughter vesicles (Figure 6A). Simultaneously, nisin exposure also triggered collapse of large mother cells and release of progeny vesicles, at a much higher rate than in the absence of nisin (Figure 6B-C, Movie S1). These findings indicate that charged mother cell membranes directly influence respiratory processes of the intracellular progeny, i.e., the energized membrane of the mother cells prevent establishment of charge and polarity among the internal vesicle membranes.

### Intracellular Vesicle Formation is Conserved in L-forms of *Listeria* and *Enterococcus*

**Figure 7:**
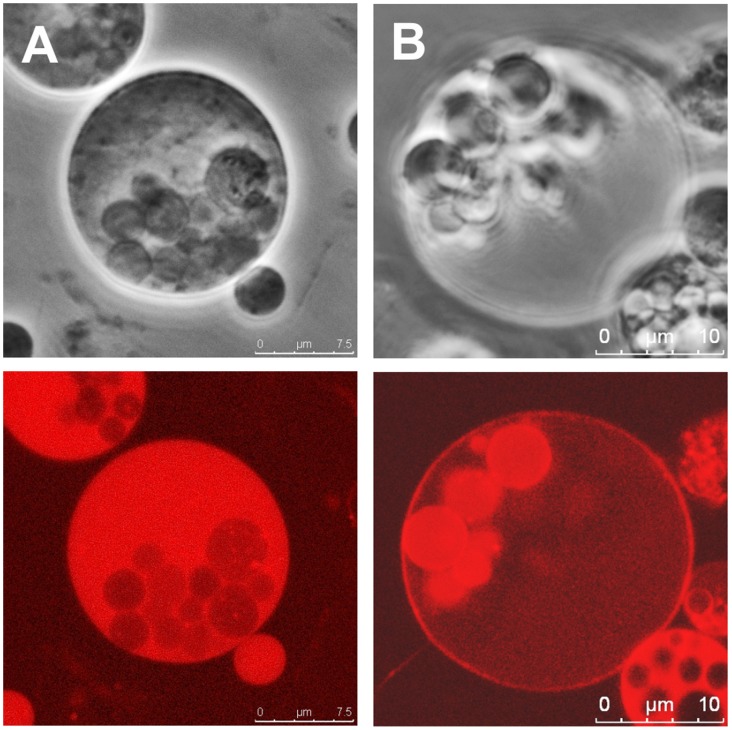
*Enterococcus* L-form cells. *E. faecium* (A) and *E. faecalis* (B) L-form cells were generated and stained with Rho123 (10 µg ml^−1^) to indicate charged membranes. In contrast to *Listeria* L-forms, the intracellular vesicles of *Enterococcus* L-forms still enclosed in the mother cell cytoplasm frequently feature stronger phase contrast (upper panels) and more intense Rho123 accumulation and fluorescence (lower panels).

An interesting aspect was to determine whether intracellular vesicle formation as a mechanism for multiplication may be more generally used, i.e., by other members of the genus *Listeria* and related bacteria. Towards this end, strains of all 8 recognized species (*L. monocytogenes*, *L. innocua, L. grayi, L. seeligeri, L. welshmeri, L. ivanovii, L. marthii,* and *L. rocourtiae*) could be successfully transformed into stable L-forms. Moreover L-form lines obtained from different members of the genes *Enterococcus* (*E. faecalis, E. faecium, E. hirae,* and *E. durans*) (Table S1, Figure S2) also showed a similar phenotype and multiplication properties. While the *Enterococcus* L-form morphology with respect to shape and arrangement is highly similar to *Listeria*, the number of intracellular vesicles per mother cell is significantly higher, and growth in soft agar medium occurs much faster. Colonies appear within 2 days, compared to 6 days with *Listeria*. Interestingly, a majority of the *Enterococcus faecium* (Figure 7A) and *faecalis* (Figure 7B) L-forms feature intracellular vesicles with high cytoplasmic density [16], which often appears darker than the mother cell cytoplasm. We also found that *Enterococcus* daughter vesicles frequently established a membrane potential (Rho123 accumulation) while still being encased by the mother cell. The larger number of intracellular vesicles, together with the higher maturation frequency inside the maternal L-form cells, may correlate to their faster growth compared to *Listeria* L-forms.

## Discussion

The L-form state by itself represents an extreme example of bacterial adaptation to specific stresses, stimuli, and/or changes in environmental conditions. The findings reported here provide insights into a highly unusual, yet apparently conserved multiplication mechanism used by *Listeria* and *Enterococcus* in their L-form status.

Transient, i.e., unstable bacterial L-forms have been described earlier. They are generally regarded as phenotypic variants, an intermediate state from which they can revert to the normal walled state. In contrast, stable L-forms are unable to reestablish an intact cell wall structure. Until now, this has been thought to be based on genetic alterations or mutations in genes directly or indirectly involved in cell wall synthesis or peptidoglycan turnover [1], [2]. In an older study from the pre-sequencing era, *Bacillus licheniformis* and *Bacillus subtilis* L-forms were reported to suffer from defective peptidoglycan precursor synthases [9]. More recent work reported mutations in the division and cell wall synthesis genes of *Escherichia coli* L-form cells [10]. Besides a frame-shift in the gene encoding MraY which catalyzes the formation of the lipid I peptidoglycan precursor, the strain also displayed additional nonsense and missense mutations in *ftsQ, ftsA, ftsW* and *murG*. In our work, resequencing of *L. monocytogenes* Scott A L-form strains subcultured for over 5 years did not yield any evidence for mutations or changes in genes involved in peptidoglycan synthesis. The L-forms featured very few polymorphisms, in a photolyase and a mannose PTS system, which are most likely not involved in cell wall synthesis or division-related processes (please see Text S1). Moreover, these changes did not occur in other stable L-form lines tested, and we conclude that these alterations are not essential for L-form transition and growth. Also, considering the ease by which L-form variants could be generated from all tested *Listeria* species and strains indicates that transition to the L-form state is based upon phenotypic plasticity, and does not require genetic predisposition or mutation-induced bottlenecks.

We here introduce techniques and methods previously not applied to investigate the nature of L-forms. These cells are highly fragile, and their analysis requires gentle manipulation and noninvasive techniques. Fluorescent staining with selective dyes and Raman microspectroscopy were very suitable for *in situ* analysis of L-forms, without the need to remove cells from their environment providing osmotic and mechanical protection. The typical colony structure of *Listeria* L-forms is characterized by slow growth at the peripheral sections, resulting in sequential appearance and accumulation of several L-form generations, which explains the unsynchronized growth and heterogeneous morphology of L-form cells within a colony. Confocal microscopy and Raman microspectroscopy both allowed single cell analysis and enabled us to study L-form developmental heterogeneity.

Although time-lapse imaging of a full reproductive cycle of L-forms falls beyond the feasibility of life cell imaging due to their slow growth [8], distortion of the membrane by pore forming peptides accelerated the process and facilitated recording of the key step, i.e., release of the progeny cells and their activation. Our findings provide additional experimental evidence for the hypothetical intracellular budding mechanism, and allow further refinement of the model. *L. monocytogenes* L-forms frequently show spatial accumulation of lipid material (Figure 2). This excess of phospholipids might be directly linked to the drastically decreased surface-to-volume ratio of L-form cells, as a result of rod-to-sphere transition and subsequent three-dimensional spherical growth [17], [18]. Phospholipid synthesis rate can be assumed to be volume- or mass-dependent, and the large spherical L-form cells insufficient surface area to incorporate all newly made phospholipids. In this context, our observation that the small daughter lipid vesicles were always immediately adjacent and in physical contact with the phospholipid accumulations suggest that the latter act as nucleation sites for the genesis of new vesicles.

A prerequisite for viability independent of the mother cells environment is that the daughter vesicles must obtain all required and essential cellular components during their genesis. Clearly, composition of this cytoplasmic “seed material” obtained during initial intracellular compartmentalization is crucial for development or fate of the vesicles. The large degree of variability observed during in the vesicle formation suggests that this process is only poorly coordinated, and distribution of components into the vesicles appears to be random rather than controlled by specific cellular programs. Multinucleation in L-forms may be regarded as a prerequisite for transition of a complete copy of chromosomal DNA into at least some of the emerging vesicles, at the time of their generation or shortly thereafter. Other essential macromolecules and components, all significantly smaller than a chromosome, are also likely encapsulated during vesicle genesis. However, the precise mechanisms and processes involved in vesicle formation and daughter cell enlargement are still unclear.

We hypothesize that the daughter cells grow in size by extravesicular addition of phospholipids, thereby expanding the initial “seed material”. Daughter vesicles are initially “silent”, and need to establish a membrane potential in order to initiate transport functions and associated metabolic activity. This is a dramatic difference and contrast to classical binary fission, in which the two progeny cells continuously maintain the existing membrane potential during and after equal division of the cytoplasmic compartment. In some way, the process in L-form cells may be reminiscent or even related to what occurs in bacterial persister cells, which can survive long starvation periods with no or an extremely low membrane potential, and are able to re-activate metabolism upon sensing suitable conditions [19], [20]. L-form daughter vesicle activation is accelerated by (and may even be dependent on) membrane depolarization of the mother cell, as demonstrated by using pore-forming peptides. The respiring mother cell may counteract or prevent establishment of a membrane charge in intracellular progeny vesicles, whereas collapse of the mother cell suddenly relieves these conditions.

A more thorough understanding of the multiplication properties featured by cell wall-deficient L-form bacteria offers a unique top-down approach, which might help to elucidate the hypothetical reproduction mechanisms of primordial cells [21]. Our findings demonstrate that L-form cells represent membrane-enclosed compartments in which metabolic key processes occur, and that an obvious lack of coordination between chromosome replication and the usually tightly regulated cytoplasmic division processes results in multinucleated cells. It is not entirely speculative that some aspects of the L-form behavior resemble the physicochemically driven membrane dynamics observed with giant lipid vesicles, which have been designed and employed as experimental models to study primitive cells and hypothetical early life forms [22]. However, a clear difference is that L-form bacteria still are equipped with the complex biochemical machinery from contemporary, evolutionary developed prokaryotic cells. They continuously produce membrane phospholipids required for vesicle enlargement and multiplication. We hypothesize that the driving force for intracellular budding in L-forms from *Listeria, Enterococcus* (and very likely other related bacteria) is the balance between osmotic regulation and excessive, poorly controlled phospholipid synthesis. By analogy, it has been shown that the generation of intravesicular vesicles inside self-reproducing giant vesicles is also dependent on intravesicular amphiphile synthesis, which results in enlargement (“swelling”) of the small non-unilamellar vesicles and membranes [23], [24].

We propose that the intracellular budding mechanism relies on simple membrane dynamics, without the need for specific, organized protein-dependent cytoskeletal arrangements or structures. Fundamental aspects, such as chromosome segregation and compartmentalization, phospholipid synthesis as driving force for vesicle reproduction, and lack of structural organization in relation to simple life forms need further and detailed elaboration. Stable L-form cells unable to revert to the parental state offer a fascinating top-down approach to study putative primordial reproduction mechanisms.

## Materials and Methods

### Induction of Cell Wall-deficiency

L-forms were generated by a two-step protocol, modified from previous procedures [8], [25]–[26]. Parental bacteria were grown for 18 h at 30°C in brain heart infusion (BHI) broth (Biolife, Milano, Italy). The culture was diluted 1/20 in hypertonic *Listeria* L-form medium (LLM; 37 g BHI broth (Biolife, Milano, Italy), 150 g sucrose, 2.5 g MgSO_4_×7H_2_O, 3.0 g whey powder (Emmi, Switzerland) per 1 liter distilled water, and grown for additional 2 hours at 30°C. Then, 50 µl volumes were plated on LLM agar plates (LLM broth plus 1.5% agar; Roth, Karlsruhe, Germany) supplemented with 50 µg ml^−1^ penicillin G (Sigma-Aldrich, St. Louis, MO). The plates were sealed with parafilm and incubated at 32°C. L-forms of *L. monocytogenes* appear within 2–4 weeks as small mucoid colonies, with a characteristical “fried egg-like” morphology. In a second step, a small amount of colony material was stabbed with a sterile needle into LLM soft agar (0.3% w/v) tubes, containing 50 µg ml^−1^ penicillin G, followed by incubation at 32°C. Spherical, vesiculated L-forms grew in distinct colonies in the LLM soft agar tube. Serial passaging in LLM soft agar with decreasing penicillin G concentrations (3×50 µg ml^−1^; 3×25 µg ml^−1^ and 3×12.5 µg ml^−1^) resulted in stabilized L-forms, which could not revert to parental bacteria when grown without drug selection. This procedure could also be shortened by direct inoculation of 100 µl of an overnight culture (grown at 30°C in LLM broth) in LLM soft agar tubes with 50 to 100 µg ml^−1^ penicillin G. Spherical and vesiculated L-forms appeared within 1–3 days. Stabilization was again achieved by repeated subcultures in LLM soft agar with decreasing amounts the drug. We found that Penicillin G could also be replaced by cephalosporidine (Sigma-Aldrich, St. Louis, MO), vancomycin (Sigma-Aldrich), and D-cycloserine (Sigma-Aldrich), providing alternatives to generate L-forms from PenG-insensitive bacteria (e.g. *L. rocourtiae* sp *nov.*) (Table S1).

### Determination of Chromosome Copy Number

In order to obtain single L-form cells, *L. monocytogenes* Scott A L-forms were generated in LLM with PenG, solidified with 2% w/v gelatin (Merck, Whitehouse Station, NJ) instead of agar. After 4 weeks, mature L-form cells could be collected by melting the gelatin at 37°C for 15 min, and harvesting the cells by gentle centrifugation (1000×g, 20 min). The tiny pellets were resuspended in 100 µl LLM broth. Then, two microliter of each of the samples were transferred to an microscope counting chamber (Helber chamber, ruling/grading 1/400 mm^2^, cell depth 0.1 mm), and eight 4×4 squares were analyzed per sample to calculate the number of cells per ml.

To prepare samples for quantitative real-time PCR (qPCR), 20 µl of the suspension was treated with DNase to remove any extracellular DNA. Then, samples were heated at 95°C for 10 minutes, frozen at −80°C to inactivate DNase and release the DNA from broken cells, and diluted fivefold in water. Quantification of the total number of *actA* gene copies per tube was performed using qPCR (Rotor Gene 6000, Corbett Robotics, Qiagen, Germany), with primers ActA-F (5′-AAGTGGCGAAAGAGTCAGTTGC-3′) and ActA-R (5′-ACTTTTAGGGAAAAATGGTTGT TGGT-3′) and SYBR Green PCR Master Mix (Life Technologies, Carlsbad, CA). Amplification and melting curve analysis was performed using the following protocol: pre-incubation (95°C for 10 min); amplification (50 cycles): 10 s at 95°C; 15 s at primer-specific annealing temperature; 20 s at 72°C, with a single fluorescence measurement and melting curve analysis from 50°C to 95°C at 2°C s^−1^ with continuous measurement [8]. As a reference, the copy number per cell of parental *L. monocytogenes* Scott A cells was determined. Here, chromosomal DNA was purified using the DNeasy tissue kit (Qiagen) according to the manufacturer's instructions.

### Genome Sequencing

L-form colonies were removed from the LLM soft agar (no antibiotics), and mixed with PBS 0.01% (v/v) Tween20 (1∶2) for osmotic lysis. Genomic DNA from the lysate was purified by phenol:chloroform:isoamylalcohol (25∶24:1) extraction, followed by ethanol precipitation and resuspension of the DNA in TE pH 7.5. Contaminating RNA was removed by using DNase-free RNase (Fermentas GmbH, St. Leon-Rot, Germany) at a final concentration 100 µg ml^−1^. Sequencing was carried out at GATC GmbH (Konstanz, Germany). A paired end library (200 bp fragments) of the purified L-form DNA was constructed, and sequencing was performed on an Illumina Genome Analyzer II. A total of 2,799,736 pairs (2×31nt) were produced, accounting for 173,583,632 bp. We used CLC Genomics Workbench 4.6.1 (CLCbio, Aarhus, Denmark) to assemble and analyze the data. Finally, the the L-form sequence was aligned with and compared to the previously determined genome of the parental *L. monocytogenes* Scott A [11].

### Confocal Fluorescence Microcopy

All images were acquired using a Leica TCS SPE confocal microscope (Leica Microsystems GmbH, Wetzlar, Germany), equipped with 405, 488, 532 and 635 nm semiconductor lasers, and operated by the Leica LAS AF interface. Sample incubation temperature was controlled at 30°C, using an incubation chamber permanently attached to the microscope (“The Cube”, Life Imaging Services, Basel, Switzerland). We used ACS APO 63x/1.30 or HCX PL FLUOTAR 100x/1.30 oil-immersion objectives. Transmission light images were obtained using Nomarski optics (DIC, Differential Interference Contrast), and phase contrast, respectively. Pinholes were always set to 1 Airy, to obtain strictly confocal images. When using multiple dyes with overlapping emission spectra, parameters were optimized to avoid cross-talk. All parameters used for the different fluorophores are listed in Table S2. Size measurements, fluorescence quantification and processing were performed with the same Leica LAS AF software. CorelDRAW X4 (Corel Corporation, Ottawa, Canada) was used for final image assembly and contrast/brightness adjustments.

Because of their fragile nature, L-form colonies can only be manipulated in a gentle manner. Centrifugation steps were largely avoided to maintain integrity of the cells. For untreated samples, colonies were directly transferred from the LLM soft agar to a glass slide and kept embedded in the soft agar for osmotic protection during analysis. For staining samples with dyes, two µl of a 10-fold concentrated dye solution (diluted in LLM broth) was mixed with 18 µl LLM soft agar containing a few colonies. After staining, unbound dye was removed by washing twice in 1 ml LLM broth, with gentle inversion and manual transfer of the colonies (except when staining with Rho123 in order not to disturb the equilibrium). A stained colony resuspended in hypertonic LLM broth (osmotic stability) was then transferred to a glass slide and carefully covered with a glass cover slip. The following stain/dyes were used: 2 µM CellTrace BODIPY methyl ester for 15 min; (Invitrogen, Carlsbad, CA), 0.5 mM 5-cyano-2,3-ditolyl tetrazolium chloride (CTC; 24 h; Sigma-Aldrich, St. Louis, MO), or 5 µg ml^−1^ rhodamine-123 (Rho123; 30 min; Invitrogen), all at room temperature. Green fluorescent protein (GFP) expressing and cytosolic fluorescent L-forms were derived from *L. monocytogenes* Scott A::pPL3-GFP [8].

The pore-forming peptide lantibiotic nisin (Sigma-Aldrich) was dissolved in 0.05% (v/v) acetic acid (pH 3.3), and stored at −20°C. One microliter of a 10-fold working stock was gently mixed with 9 µl LLM soft agar containing a few L-form colonies. Final nisin concentration was 0.625 µg ml^−1^ (or 185 nM). The samples were incubated for 5 minutes at room temperature, followed by 1×10^−4^ dilution in LLM broth, to prevent nisin from reaching the intracellular vesicles which are released approximately 10–15 minutes after nisin challenge. Colonies and single cells were then observed using confocal microscopy and time-lapse imaging.

### Confocal Raman Microspectroscopy

A confocal LabRAM HR800 system (Horiba, Darmstadt, Germany) equipped with a 532.17 nm laser was used to determine the intracellular components of L-form cells. Colonies removed from soft agar tubes were carefully washed twice with 1 ml protoplast buffer (1 M sucrose, 130 mM NaCl, 50 mM Tris-HCl and 10 mM MgCl_2_) to remove residual soft agar. Colonies were then pipetted onto a CaF_2_ slide, and covered with a normal glass cover slip. The cells were kept in protoplast buffer during the measurement, which explains the background signals. First, cells were selected for spectral recordings in the live-view mode of the Labspec software (Horiba, Darmstadt, Germany), and brought into focus using the fluorescent signal. Individual target cells were manually aligned with the blue spot where the Raman laser beam would hit the sample. For Raman analysis, the selected cells were exposed to the beam for 30 s, with 3 accumulations. A 100x/0.9 objective (laser spot <1.2 µm according to the Rayleigh criterion) was used, and the pinhole was set to 100 µm, which corresponds to an optical slice thickness of 1.84 µm. Intracellular vesicles with a diameter of at least twice the optical slice thickness were selected, and those were hit exactly in their middle. A D1 intensity filter was applied to prevent burning of the cells. Raman spectra were acquired between 550 and 1750 cm^−1^, which includes most peaks reflecting biological molecules. Raman spectra were baseline corrected, normalized and exported to a file format readable by Excel (Microsoft).

## Supporting Information

Figure S1 **Colony structure.** A single L-form colony grown in hypertonic soft agar was imaged at low resolution using a 10x/0.3 objective lens. To obtain higher resolution, images taken with a 63x/1.3 objective lens were stitched together. The inlay shows a digital zoom up to 150x. A tremendous variation in morphology can be observed among an L-form population. The core of the colony is composed of cell debris of previous generations, while young, actively growing L-forms are located at the border of the colony. L-forms do not only differ in size, a subpopulation also contains between one and up to several tens of membrane vesicles. L-forms with intracellular vesicles (marked with arrow heads) are mainly located in the peripheral zone.
(TIF)

Figure S2 **Induction of** ***Listeria*** **and** ***Enterococci*** **species to the L-form state.** All *Listeria* (A) and *Enterococci* (B) are directly induced from parental cells (without a ‘fried egg-like’ intermediate, see above) under the conditions described in Table S1. The upper panels depict the parental cells, the lower panels show induced L-forms.
(TIF)

Table S1 **Overview of transformed species and conditions.** Eight *Listeria* species and four *Enterococcus* species could be transformed to the L-form state. The used antibiotic (penicillin G, cephalosporidine, vancomycine and D-cycloserine are abbreviated as Pen, Cep, Van and Cyc, respectively) and concentrations (in µg ml^−1^) are indicated. Most of them could be repeatedly subcultivated, were actively growing and exhibited similar morphological characteristics. *L. grayi* subsp. *murayi* WS2283 L-forms needed a higher sucrose and MgSO_4_ concentration to be stabilized. In addition, Pen50 and Cep50 resulted only in a partial transition of the inoculum, while Van50 led to complete transition. Further subcultivation of *L. grayi* subsp. *murayi* WS2283 L-forms was unsuccessful and no multiplication was observed. *L. rocourtiae* sp. *nov.* [27] is penicillin resistant, but Van100 and Cyc500 were successfully applied for L-form transition. Some minor growth after subcultivation was observed for Van100, but they could not be continuously passaged. *E. durans* DSM20633T could be partially induced to the L-form state (mixed culture of large L-forms and enlarged, more spherical parental cells), but no L-form reproduction was observed. Species which could not be induced to the L-form state with our protocol include *E. avium* DSM20679, *Bacillus subtilis* 168, *B. subtilis* ATCC9372, *Bacillus cereus* DSM360, *Streptococcus agalactiae* GD9, *Staphylococcus aureus* ATCC6538, *Staphylococcus epidermidis* DSMZ 1798, *Lactococcus lactis* [28], *Lactobacillus casei* ATCC334 and *Escherichia coli* K-12.
(XLSX)

Table S2 **Specific settings for each fluorophore.** Excitation wavelength and emission spectrum are given for each fluorophore for single and double labeling experiments.
(XLSX)

Text S1 **Resequencing of stable L. monocytogenes Scott A L-forms.** The text describes the additional methods used, and provides detailed results and a short discussion.
(DOC)

Movie S1 **Refers to** **Figure 6B** **.** Brief exposure to 0.625 µg ml^−1^ nisin depolarizes the mother cell membrane potential, often resulting in a sudden collapse of the mother cell. The DIC- (left) and GFP-channel (right) are shown. The time scale is shown in the upper right corner.
(MP4)
