# Supplementary figures and images for: Intracellular Vesicles as Reproduction Elements in Cell Wall-Deficient L-Form Bacteria
Source: PLoS One. 2012 Jun 6;7(6):e38514. doi: 10.1371/journal.pone.0038514 (PMC3368840; doi:10.1371/journal.pone.0038514)

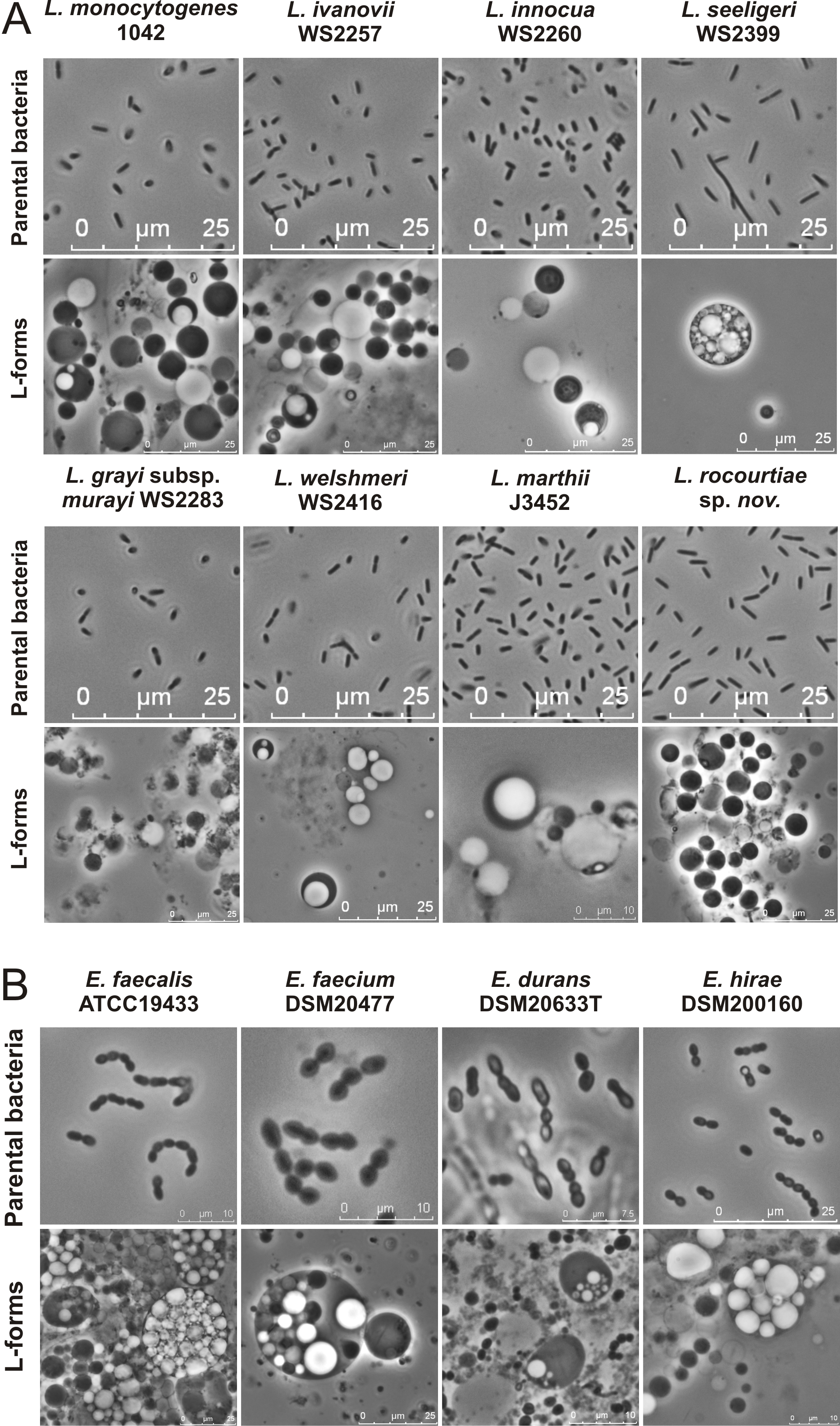

Supplement: Figure S2 — Induction of Listeria and Enterococci species to the L-form state. All Listeria (A) and Enterococci (B) are directly induced from parental cells (without a ‘fried egg-like’ intermediate, see above) under the conditions described in Table S1. The upper panels depict the parental cells, the lower panels show induced L-forms. (TIF) [file pone.0038514.s002.tif]
