# Supplementary material for: Intracellular Vesicles as Reproduction Elements in Cell Wall-Deficient L-Form Bacteria
Source: PLoS One. 2012 Jun 6;7(6):e38514. doi: 10.1371/journal.pone.0038514 (PMC3368840; doi:10.1371/journal.pone.0038514)
Supplement: Text S1 — Resequencing of stable L. monocytogenes Scott A L-forms. The text describes the additional methods used, and provides detailed results and a short discussion. (DOC) [file pone.0038514.s005.doc]

***Supporting Information***

**Intracellular vesicles as unique reproduction elements in cell wall-deficient bacteria**

Yves Briers, Titu Staubli, Markus C. Schmid, Michael Wagner, Markus Schuppler,
and Martin J. Loessner

**Resequencing of stable *L. monocytogenes* Scott A L-forms**

The sequence reads of the Scott A L-form genome were mapped against the reference genome of parental Scott A (GenBank CM001159 ) to identify single nucleotide polymorphisms (SNPs), deletions, insertions or other polymorphisms. All known peptidoglycan synthesis and cell division related genes were completely conserved in the L-form genome. Complete comparison of the assembled genomes of parental bacteria and L-forms revealed two SNPs and one 34 nt deletion. One of both SNPs and the deletion were located in an open reading frame, whereas the second SNP is located 51 bp upstream the start codon of an open reading frame. All reads covering these polymorphisms were specific and could not be matched elsewhere in the genome. PCR primers were designed to amplify the regions flanking the different polymorphisms and subsequent Sanger sequence analysis. Both SNPs and the DIP were confirmed.

A deletion of 34 nt between bp 707 and 740 of the open reading frame encoding hydroxymethylglutaryl-CoA synthase (HMG-CoA synthase; *lmo1415*; total length 388 amino acids) results in a modified amino acid sequence from amino acid 235 and a premature stop codon after 248 amino acids. The fragment of 34 nt is possibly excised by a single homologous recombination of an imperfect 6 bp repeat (TACCAT-N_28_-TACTAT). HMG-CoA synthase is part of the mevalonate-dependent isoprenoid pathway. Isopentenyl diphosphate (IPP) is the central intermediate in the biosynthesis of isoprenoids. The latter are the most ancient and diverse class of natural products and serve numerous biochemical functions for respiratory energy generation (e.g. quinones), oxidative stress protection (e.g. heme groups) and peptidoglycan precursors (bactoprenol). Two distinct and independent pathways to IPP exist in *Listeria monocytogenes* (see figure below). The mevalonate-dependent route starts from acetyl-CoA and proceeds through the intermediate mevalonic acid. HMG-CoA synthase condenses acetyl-CoA with acetoacetyl-CoA to form 3-hydroxy-3-methylglutaryl-CoA (HMG-CoA), which is further reduced to mevalonate by HMG-CoA reductase. An alternative biosynthetic route is the non-mevalonate 2-C-methyl-D-erytritol 4-phosphate (MEP) pathway. It has been shown that each pathway is functional but dispensable for viability in *Listeria monocytogenes* and that disruption of the mevalonate-pathway leads to a complementary upregulation of the MEP pathway .

Acetyl-CoA

Acetoacetyl-CoA

HMG-CoA

Mevalonate

Mevalonate-P

Mevalonate-PP

GA3P + Pyruvate

DOXP

MEP

CEP-ME

CDP-MEP

MEcPP

Isopentenyl diphosphate (IPP)

Mevalonate pathway

MEP pathway

HMG-CoA synthase

Isoprenoids (including bactoprenol)

HMB-PP

**Fig. 1** Mevalonate and MEP pathway for isoprenoid synthesis in *Listeria monocytogenes.*

Different intermediates and the enzymatic function of HMG-CoA synthase are shown. Abbreviations: HMG-CoA, 3-hydroxy-3-methylglutaryl-coenzyme A; GA3P, glyceraldehyde-3-phosphate; DOXP, 1-deoxy-D-xylulose 5-phosphate; MEP, 2-C-methyl-D-erytritol 4-phosphate; CDP-ME, 4-diphosphocytidyl-2-C-methyl-D-erythritol; MEcPP, 2-C-methyl-D-erythritol 2,4 cyclopyrophosphate ; HMB-PP, (*E*)-4-hydroxy-3-methyl-*but*-2-enyl pyrophosphate.

A point mutation was located at position -51 upstream from the start codon of the first open reading frame (*lmo0096*) of an operon of three genes, encoding the IIAB, IIC and IID component of the putative mannose-specific phosphotransferase system, respectively. It is unclear whether this mutation has an immediate impact on transcription regulation of the operon. Array-based transcriptomics data of Scott A L-forms showed previously that metabolism-related genes and genes encoding ribosomal proteins are generally downregulated in Scott A L-forms . Expression of the two last genes of the operon (encoding components IIC and IID) are strongly down-regulated (16.4 and 34.5x, respectively). Also genes encoding components of other sugar phosphotransferase systems are downregulated (fructose specific PTS IIABC component 2.6x; lichenan specific PTS IIA component 3.3x; cellobiose specific PTS IIB component 24.5x; cellobiose specific PTS IIA component 9.5x; cellobiose specific PTS IIC component 14.3x; PTS phosphocarrier protein Hpr 3.9x).

A missense mutation in codon 980 of the gene coding for deoxyribodipyrimidine photolyase (*lmo0588*) results in the replacement of a proline by a serine residue. This enzyme is involved in DNA repair after UV radiation. Cyclobutapyrimidine is resolved to two pyrimidine units . The gene expression of two other genes whose gene products is involved in DNA repair are downregulated in Scott A L-forms: *lmo2676* (UV damage repair protein 8.7) and *recU* (similar to DNA repair and homologous recombination protein 2.5).

Continuous subcultivation of stable L-form cultures is done by collecting the whole culture content, dilution, splitting and transfer of a portion to fresh tubes. This technique has probably prevented the accumulation of more mutations. Spontaneous mutations which appear in single colonies would much more easily accumulate if only single colonies would be transferred.

Polarization effects caused by one or more of found mutations cannot be excluded, however, functional analysis of downstream located genes does not indicate such effects as a possible explanation for L-form stabilization. Altogether, we conclude that L-form stabilization is here not caused by genotypic changes.

**Supplementary References**

Begley M, Gahan CG, Kollas AK, Hintz M, Hill C, et al. (2004) The interplay between classical and alternative isoprenoid biosynthesis controls gammadelta T cell bioactivity of *Listeria monocytogenes*. FEBS Lett 561: 99-104.

Briers Y, Klumpp J, Schuppler M, Loessner MJ (2011) Genome sequence of *Listeria monocytogenes* Scott A, a clinical isolate from a foodborne listeriosis outbreak. J Bacteriol 193: 4284-4285.

Dell'Era S, Buchrieser C, Couvé E, Schnell B, Briers Y, et al. (2009) *Listeria monocytogenes* L-forms respond to cell wall deficiency by modifying gene expression and the mode of division. Mol Microbiol 73: 306-322.

Weber S (2005) Light-driven enzymatic catalysis of DNA repair: a review of recent biophysical studies on photolyase. Biochim Biophys Acta 1707: 1-23.
